# Supplementary material for: The co-evolution of multiply-informed dispersal: information transfer across landscapes from neighbors and immigrants
Source: PeerJ. 2013 Feb 26;1:e44. doi: 10.7717/peerj.44 (PMC3628985; doi:10.7717/peerj.44)
Supplement: Supplemental Materials [file peerj-01-44-s001.doc]

**SUPPLEMENTAL ONLINE MATERIALS**

**Determining ‘Evolution’ of a Strategy**

All individuals were capable of each form of information use, but the utility and benefit of this strategy would depend on the influence that this information had on dispersal. In other words, individuals might be capable of informed dispersal, but will only adopt that strategy if the coefficient was high enough that environmental conditions lead to a high probability of dispersal. Therefore, unless the entire metapopulation went extinct, all strategies necessarily existed in the population. Empirical investigations in the common lizard suggest that individuals indeed use multiple sources of information in dispersal, although each individual may prioritize information differently (Le Galliard et al. 2003; Cote & Clobert 2007b; Cote et al. 2008). We determined that evolution of an informed dispersal strategy had occurred using two methods. First, we examined if the evolved coefficient was greater than 0 more often than by chance since drift should lead to negative coefficients as often as positive ones. In all cases, dispersal coefficients were significantly more often positive (Sign test: D-only P<0.0001; I-only P<0.0001; D&I: I evolution P<0.0001, D evolution P=0.035). Therefore, in figures we considered a behavior to have evolved if its coefficient was positive (i.e. *D* or *I* > 0). Second, we used an alternative method to detect the evolution of dispersal coefficients by identifying runs where the partial dispersal rate attributable to that strategy increased above background values. We determined background noise values by quantifying the random variation in dispersal on the fixed uninformed dispersal strategy (*d*_U_ = 10%). The variance in the dispersal rate of U was 0.8% around the mean of 10% dispersal, so we designated an informed strategy as having ‘evolved’ if it attained dispersal rates greater than 1% above baseline. Joint evolution of the two informed strategies in models where both were present included simulation runs where dispersal by both D and I were each 1% above baseline values. Baseline values depend on the fixed intercept of the informed dispersal equations as described below (see section on *Intercepts* below). Results using this method are very similar to those counting cases where the coefficient was positive and so the first method was used in all figures. We also re-did evolutionary analyses by designating cases of ‘evolution’ only when the dispersal rate was 5% above baseline, and all general patterns were again similar despite a small quantitative difference in the probability of evolution. The similarity of results is most likely due to the fact that when a behavior does evolve, it nearly always leads to larger coefficients and a partial dispersal rate well above 15%.

**Additional Model Explorations**

We examined a range of values for other parameters that have well known effects on dispersal and metapopulation dynamics in the theoretical literature to examine the generality of our model. These model explorations examined 1) the effects of life history parameters (fast vs. slow), 2) the effects of patch number, 3) the effects of patch carrying capacity, 4) the effects of environmental stochasticity, 5) the effects of dispersal costs, 6) the influence of the fixed intercept in information-dependent dispersal equations, 7) the effects of a fixed uninformed dispersal rate, 8) the effects of evolving uninformed dispersal, 9) the effects of the order in which different sources of information are used, and 10) how immigrant-dependent information is processed.

*Life History Parameters*

We simulated two different life history strategies to examine the effect of variation in survival and reproduction on mean dispersal rates. For most parameter combinations, we used a ‘fast’ life history roughly equivalent to a small lizard or passerine life cycle (survival: s_0_=0.2, s_1_=0.35, s_2_=0.5; fecundity: f_1_=7, f_2_=7). We also compared these results with a ‘slow’ life history roughly equivalent to an ungulate life cycle (survival: s_0_=0.7, s_1_=0.8, s_2_=0.85; fecundity: f_1_=0, f_2_=1). These parameters were chosen to produce identical population growth rates of fast and slow life cycles in the absence of density regulation. The average dispersal rate was higher for the fast life history than the slow life history (0.59 vs. 0.12 respectively). However, life history had little influence on the probability that either form of informed dispersal evolved on its own and nearly no effect on the probability that the two coexisted (Fig. S1).

*Patch Number*

The number of patches had little influence on the probability that informed dispersal evolved for either information use strategy alone or on the coexistence of both strategies when both were possible (D&I; Fig. S2). While populations with 5 patches had a slightly lower rate of coevolution (10% less), the two larger metapopulations showed no difference.

*Carrying Capacity*

Very small carrying capacities (<50) lead to much higher rates of metapopulation extinction (Fig. S3), however the extinction rate was largely influenced by information use (see main text; Fig. 5d) since use of multiple sources of information to disperse generally reduced extinction risk. Variation in carrying capacity above levels where extinction rates were high (*K* = 50, 100, 200), had a little influence on the probability that immigrant dependent dispersal evolved. Density dependent dispersal evolved somewhat more often as the carrying capacity increased. In contrast, the coevolution of both density- and immigrant-dependent information use decreased when the population carrying capacity increased (Fig. S4).

*Environmental Stochasticity*

Environmental stochasticity should lead to increased heterogeneity in population sizes among patches and thus increase the benefits of dispersal from high density patches. We introduced different levels of environmental stochasticity by defining the fraction of patches that would get hit at each time step and the decrease in survival for all individuals in those populations that are affected. We varied environmental stochasticity from low (patches affected per time step: 1%) to high (patches affected per time step: 10%) frequency and reduced survival of all individuals in affected patches by 20%. Increasing environmental stochasticity from low to medium levels (1-5%) greatly increased the benefits of dispersal (Fig. 5a-c, S5), but did not have major effects on the average dispersal rate. Increased environmental stochasticity did lead to a greater probability of metapopulation extinction as expected, and this rate of extinction was slightly lower in models with use of one or more sources of information (D only, I only, or D&I) in dispersal decisions (Fig. 5c). This lower population extinction rate mirrors the lower extinction rate for models with multiple sources of information as patch carrying capacity decreases. Once we control for the increased probability of metapopulation extinction, the probability that both forms of information use coevolved showed little consistent pattern among persisting populations in all models (D&I). While higher levels of stochasticity that did not always lead to complete extinction (between 5%-3%) also had a higher rate of coevolution, so few trajectories survived that the differences may not be robust. Once most trajectories survived (between 3-0%), the rate of coevolution was fairly constant.

*Costs of dispersal*

Because costs of dispersal could greatly influence the evolution of information use (Travis et al. 1999; Bonte et al. 2012) we also explored situations where informed dispersal was costly. The cost of dispersal decreased the probability that dispersers survived and depended on the type of information used in the dispersal decision. We made the basic assumption that the use of immigrant information should usually reduce costs of dispersal relative to no information or information about local population density only. The arrival of immigrants proves that habitable populations are attainable and thus reduces the risk that a disperser will not find a suitable patch whereas local density or uninformed dispersal contains no insight about the feasibility of finding another patch. Therefore, density dependent dispersal is expected to carry greater survival costs than immigrant dependent dispersal.

The cost of dispersal did affect the evolutionary dynamics of each strategy (Fig. S6, S7), but did not completely prevent the evolution of informed dispersal nor the coevolution of the two behaviors (Fig. S7). The probability that informed dispersal evolved declined similarly with increasing costs of dispersal for both density and immigrant information sources (Fig. S6). When costs of each informed strategy differed significantly, coexistence of information use and benefits of informed dispersal from two sources was maintained over very long time scales (Fig. S7) despite a considerable decline in the probability that both forms of information use evolved simultaneously (Fig. S7). When the cost of density-dependent dispersal was 5% higher than that of immigrant-dependent dispersal, the probability that both behaviors coevolved remained, but at a low level not significantly above drift (13% of simulations with up to 32% expected by drift; Fig. S7). Overall, coexistence of information use in dispersal from both direct and indirect sources using very simple rules of thumb occurred even when costs of using different forms of information were asymmetric.

*Intercepts*

We included intercepts for the equations of information dependent dispersal (eqns 1 and 2) so that baseline levels of dispersal when information was not present (i.e. when density was near zero and there were no immigrants) resulted in levels of dispersal near zero percent. We tested two different intercepts that resulted in baseline dispersal of 2% and 12% (intercepts of -4 and -2 respectively). A lower intercept (-4) required a much larger coefficient to evolve before information dependent behavior had a significant effect on dispersal rates. This resulted in much longer times before coefficients stabilized and led to a lower probability that both density and immigrant dependent dispersal coevolved (Fig. S8). However, both behaviors still coevolved in a significant proportion of simulations.

*Fixed uninformed dispersal*

The exclusion of a fixed baseline uninformed dispersal rate (*d*_U_ = 10%) in our basic model had very little effect on the equilibrium mean dispersal rate of the metapopulation and had little impact on the co-evolution of density and immigrant-dependent dispersal (Fig. S9, compare ‘Fixed U’ vs. ‘No U’). Indeed, this would be expected since the evolution of dispersal *per se* should be driven primarily by the benefits of dispersal rather than the means by which this rate is achieved. The presence of a fixed baseline uninformed dispersal had a different effect on the probability that either density dependent or immigrant dependent information use evolved when either was the only source of information available (Fig. S10). Immigrant-dependent dispersal depends on the arrival of immigrants and so could not evolve without some baseline values. In contrast, the evolution of density-dependent dispersal was unaffected by the presence of background dispersal when it was the only source of information available. Overall, the presence of a fixed uninformed dispersal rate had little effect on the co-evolutionary dynamics of informed dispersal despite being essential for immigrant-dependent dispersal to evolve alone.

*Evolving uninformed dispersal*

Competition from uninformed dispersal could nullify the benefits of informed dispersal if uninformed dispersal could achieve optimal levels of dispersal for that metapopulation. We allowed uninformed dispersal to freely evolve to any value in competition with informed dispersal. Overall, competition from evolving uninformed dispersal did not impede the evolution of informed dispersal, but did reduce the probability that informed dispersal evolved. The actual impact of competition from U depended on the type of information used. Competition with just a single source of information caused a reduction in the probability that informed dispersal evolved and this effect was similar for density- and immigrant dependent dispersal (16% and 15% respectively, Fig. S10). Competition with multiple sources of information (D&I model) also led to a slight decrease in the probability that both sources of information coevolved (14% decrease, Fig. S9). The evolutionary dynamics were little affected by competition with evolving uninformed dispersal.

*Order of Information Use*

Potential competition from two different information sources could mean that the order in which density and immigrant sources of information are used could influence the evolution and relative importance of each strategy. Therefore, in models where both density and immigrant-dependent sources of information were available, we also examined the order in which each source of information was used by an individual. In addition to the model presented in the main text where each source of information was used simultaneously (case D&I), we also ran models where one source of information took priority over the other.

Case D→I: the local density was considered before the number of immigrants.

Case I→D: the number of immigrants was considered before the local density.

Regardless of the costs of dispersal by each strategy, the simultaneous use of information (D&I) lead to the highest probability that the two behaviors coevolved (48% compared to 30% and 44% for I→D and D→I respectively; Fig. S11). However, even in cases where the order of information use was not optimal for the coexistence of two information sources (i.e. I→D), both sources of information coexisted in a significant proportion of simulations. Immigrant-dependent dispersal showed minor differences in the frequency with which it evolved when both forms of information were available (D&I: 82%; I→D: 83%; D→I: 88%; Fig. S11). Interestingly, immigrant-dependent dispersal evolved most frequently when it was the second source of information considered. In contrast, density dependent dispersal evolved much less often when it was the second source of information considered relative to the other two information use sequences (D&I: 65%; I→D: 46%; D→I: 60%; Fig. S11). Overall, the sequence of information use affected the co-evolution of informed dispersal in some cases suggesting that the priority of different types of information use are likely to be important in natural systems.

*Indirect Immigrant-based Information Processing*

While density dependent dispersal behavior is fairly easy to model based on a rich theoretical history (∝*N/K*), immigrant dependent dispersal is a new concept lacking the empirical or theoretical background upon which to model the behavior. We initially modeled immigrant dependent dispersal as the number of immigrants arriving in a patch (∝*M*) under the assumption that immigrants stand out from the crowd and thus detection of immigrants does not depend on the number of other individuals present in the population (Eq. 2). This formulation is consistent with the two empirical investigations we are aware of (lizards: Cote & Clobert 2007a; *T. thermophila* ciliates). However, other formulations are equally valid if immigrants do not stand out from the crowd or the presence rather than the number of immigrants triggers dispersal. Therefore, we also modeled immigrant dependent dispersal as the number of immigrants relative to population size (∝*M/N*) in a patch and as the presence of immigrants in a patch (*M*>0; in Eq. 2, *M* is replaced by 1 if *M*>0, and by 0 otherwise).

The mechanism underlying immigrant dependent dispersal had very little effect on the probability that density and immigrant-dependent dispersal coevolved in models where both sources of information were used together (D&I; Fig. S12a). Coevolution of immigrant and density dependent information was remarkably similar in the basic model (∝*M*, 48%) compared to the relative contribution model (∝*M*/*N*, 46%) and immigrant presence model (*M*>0, 49%). This is all the more striking since the way in which immigrant information is used had an important impact on the proportion of cases where density- and immigrant-dependent dispersal each evolved. Density dependent dispersal evolved much more often when immigrant-dependent dispersal was scaled to population density (∝*M*/*N*; prob = 95%) relative to the other two formulations (∝*M*, 65%; *M*>0, 75%). Immigrant-dependent dispersal evolved much less often when immigrant-dependent dispersal was scaled to population density (∝*M*/*N*, 48%) relative to the other two formulations (∝*M*, 82%; *M*>0, 75%). Indeed, when immigrant number was scaled to population density, it did not evolve more than expected by drift (∝*M*/*N*, 48%; sign test P = 0.7). There was also an important influence of how we modeled immigrant information use on the probability of evolution when immigrant information was the only source of information possible (I-only; Fig. S12b). While immigrant-dependent dispersal evolved easily when immigrant number or presence was used (∝*M*, 99%; *M*>0, 97%), it evolved much less often when immigrant number was scaled to population size and did not consistently evolve relative to drift (∝*M*/*N*, 58%; Sign test P = 0.13). This may be in large part due to the fact that scaling to population size greatly reduces the impact to the coefficient and would require a much larger increase in coefficient for immigrant-information to have much impact on dispersal. Such an effect could probably be achieved with larger mutations to the coefficient.

**Figure S1:** Effect of patch number on the evolution of informed dispersal. Plotted are the proportion of simulations where dispersal evolved based on density dependent information (D, red), immigrant information (I, blue), both density and immigrant information (D+I, red and blue hatch), or where dispersal did not evolve (None, white) across 100 Monte Carlo simulations for a model with both density and immigrant dependent dispersal present (D&I).

**Figure S2:** Effect of patch number on the evolution of informed dispersal. Plotted are the proportion of simulations where dispersal evolved based on density dependent information (D, red), immigrant information (I, blue), both density and immigrant information (D+I, red and blue hatch), or where dispersal did not evolve (None, white) across 100 Monte Carlo simulations for a model with both density and immigrant dependent dispersal present (D&I).

**Figure S3:** The effects of carrying capacity and use of different sources of information on meta-population extinction using the basic model (no evol of U; coefficients and intercepts of D and I evolve). Shown is the effect of carrying capacity on the time to extinction for populations with 100% meta-population extinction.

**Figure S4:** Effect of carrying capacity on the evolution of informed dispersal. Plotted are the proportion of simulations where dispersal evolved based on density dependent information (D, red), immigrant information (I, blue), both density and immigrant information (D+I, red and blue hatch), or where dispersal did not evolve (None, white) across 100 Monte Carlo simulations for a model with both density and immigrant dependent dispersal present (D&I).

**Figure S5:** The relative benefit of dispersal behavior is estimated as how much better a disperser did by moving (i.e. old pop density/ new pop density both at reproduction) over the first 100000 time steps. Values > 1 indicate a benefit. Dispersal benefit shown for models without environmental stochasticity with A) I-only, B) D-only, or C) D&I. Note that the scale of the benefit is lower than in models with environmental stochasticity (see Fig. 4a, b).

**Figure S6:** Effect of the cost of dispersal according to each information source on the evolution of informed dispersal. Figures show the evolution of A) density dependent dispersal only, B) immigrant-dependent dispersal only, and C) joint dispersal by both density and immigrant sources of information.

**Figure S7:** Effect of the cost of dispersal on density dependent dispersal on the coevolution of density and immigrant dependent dispersal when both forms of information are present. Costs are higher for density dependent dispersal because departure from a patch with no knowledge of the presence and accessibility of other populations carries greater risks. The arrival of immigrants certifies that other populations exist and can be attained. Plotted are the proportion of simulations where dispersal evolved based on density dependent information (D, red), immigrant information (I, blue), both density and immigrant information (D+I, red and blue hatch), or where dispersal did not evolve (None, white) across 100 Monte Carlo simulations for a model with both density and immigrant dependent dispersal present (D&I).

**Figure S8:** Effect of the fixed intercept in informed dispersal equations on the evolution of informed dispersal. Plotted are the proportion of simulations where dispersal evolved based on density dependent information (D, red), immigrant information (I, blue), both density and immigrant information (D+I, red and blue hatch), or where dispersal did not evolve (None, white) across 100 Monte Carlo simulations for a model with both density and immigrant dependent dispersal present (D&I).

**Figure S9:** Effect of baseline dispersal (U) on the evolution of informed dispersal. Columns represent models where baseline dispersal was fixed, absent, or could evolve as did informed dispersal. Plotted are the proportion of simulations where dispersal evolved based on density dependent information (D, red), immigrant information (I, blue), both density and immigrant information (D+I, red and blue hatch), or where dispersal did not evolve (None, white) across 100 Monte Carlo simulations for a model with both density and immigrant dependent dispersal present (D&I).

**Figure S10:** Effect of baseline dispersal (U) on the evolution of informed dispersal. Columns represent models where baseline dispersal was fixed or could evolve as did informed dispersal. Plotted are the proportion of simulations where dispersal evolved based on immigrant information (I, blue) or density dependent information (D, red) along with cases where dispersal did not evolve (None, white) across 100 Monte Carlo simulations for models with A) immigrant information only or B) density dependent dispersal only.

**Figure S11:** Effects of the order of information on the evolution of informed dispersal. Plotted are the proportion of simulations where dispersal evolved based on density information (D, red), immigrant information (I, blue), both density and immigrant information (D+I, red and blue hatch), or where dispersal did not evolve (None, white) across 100 Monte Carlo simulations in a model with both density and immigrant dependent dispersal (D&I). Information use ‘evolved’ if the coefficient governing dispersal from that behavior was positive. Columns show models where the two forms of information are used simultaneously (D&I), when immigrant information is used first (I→D), and when density information is used first (D→I).

**Figure S12:** Effect of the immigrant-informed dispersal equation on the evolution of informed dispersal. Columns represent models where immigrant-dependent dispersal was influenced by the number of immigrants (unscaled), the proportion of immigrants in the population (scaled), or as a threshold where the first immigrant triggers a probability to disperse. Plotted are the proportion of simulations where dispersal evolved based on density dependent information (D, red), immigrant information (I, blue), both density and immigrant information (D+I, red and blue hatch), or where dispersal did not evolve (None, white) across 100 Monte Carlo simulations. Models with A) both density and immigrant dependent dispersal present (D&I) and B) Immigrant-dependent only dispersal is present (I-only).
